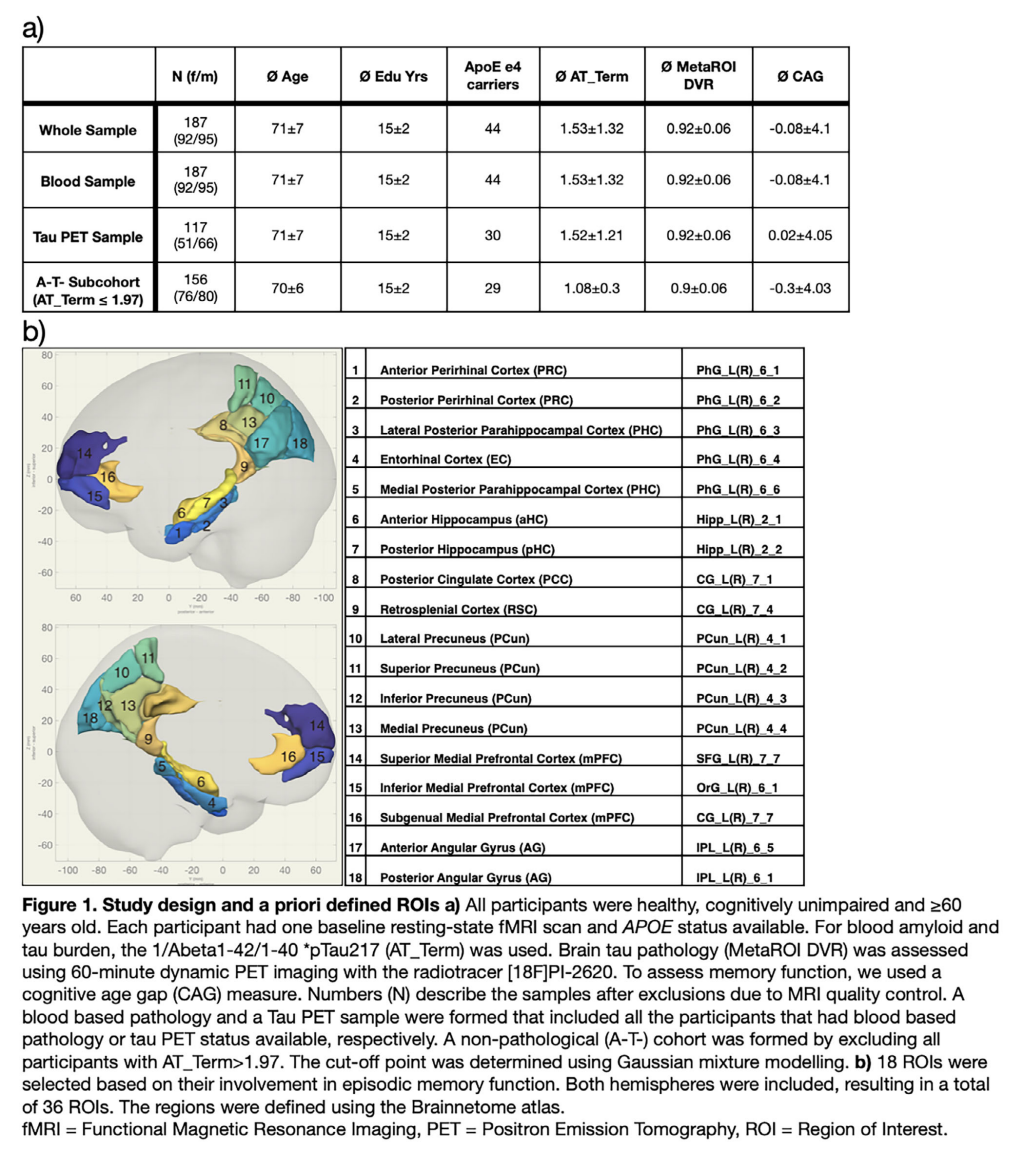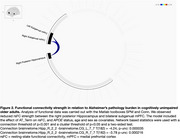# Episodic memory network connectivity with aging and Alzheimer’s disease pathology in cognitively unimpaired older adults

**DOI:** 10.1002/alz70861_108678

**Published:** 2025-12-23

**Authors:** Julia Gatzen, Larissa Fischer, Niklas Behrenbruch, Beate Schumann‐Werner, Svenja Schwarck, Eóin N. Molloy, Anne Hochkeppler, Berta Garcia‐Garcia, Anna‐Therese Büchel, Enise I Incesoy, Michael Rullmann, Marianne Patt, Andreas Schildan, Andrew W. Stephens, Gusalija Behnisch, Barbara Morgado, Hermann Esselmann, Constanze I. Seidenbecher, Björn H. Schott, Henryk Barthel, Osama Sabri, Jens Wiltfang, Michael C. Kreissl, Emrah Düzel, Anne Maass

**Affiliations:** ^1^ German Center for Neurodegenerative Diseases (DZNE), Magdeburg Germany; ^2^ University of California, Irvine, CA USA; ^3^ Institute of Cognitive Neurology and Dementia Research (IKND), Otto‐von‐Guericke University, Magdeburg Germany; ^4^ Division of Nuclear Medicine, Department of Radiology & Nuclear Medicine, Faculty of Medicine, Otto von Guericke University, Magdeburg Germany; ^5^ University Clinic for Psychosomatic Medicine and Psychotherapy, Otto‐von‐Guericke University Magdeburg, Magdeburg Germany; ^6^ Department of Nuclear Medicine, University of Leipzig, Leipzig Germany; ^7^ Life Molecular Imaging GmbH, Berlin Germany; ^8^ Leibniz Institute for Neurobiology (LIN), Magdeburg Germany; ^9^ German Center for Neurodegenerative Diseases (DZNE), Göttingen Germany; ^10^ Department of Psychiatry and Psychotherapy, University Medical Center Göttingen (UMG), Göttingen Germany; ^11^ Faculty of Natural Sciences, Otto‐von‐Guericke University Magdeburg, Magdeburg Germany

## Abstract

**Background:**

Regions of the medial temporal lobe and posteromedial cortex are crucial for episodic memory and are among the first to be affected by Alzheimer´s disease (AD) pathology. Hyperconnectivity in association with amyloid and tau pathology has been found in preclinical stages of AD, and may be compensatory or detrimental to memory function. Aiming to identify distinct connections displaying aberrant resting‐state functional connectivity (rsFC), we hypothesized lower rsFC with higher age in non‐pathological aging, and higher rsFC with higher pathology burden, especially in *APOE4* carriers.

**Method:**

In this preregistered study, we analysed cross‐sectional resting‐state fMRI data and blood‐ and PET‐markers of amyloid, tau and *APOE* status from an observational aging cohort (SFB1436). RsFC strength was examined between predefined ROIs. The sample included 187 cognitively unimpaired older adults (71±7years, 92 female, 44 *APOE4* carrier). We investigated associations between rsFC, AD pathology burden, and CAG (cognitive age gap) and potential *APOE* interactions. Multiple regression models and a moderation analysis with rsFC strength were used. An A‐T‐ subcohort was formed to investigate the effect of age on rsFC and to differentiate it from the effect of pathology.

**Result:**

We found a significant association between higher blood‐based pathology and low rsFC in the right posterior hippocampus to bilateral subgenual medial prefrontal cortex (mPFC). No significant association was observed between rsFC and tau‐PET burden, APOE4 status or age (within A‐T‐subcohort). Additionally, there was no significant effect of pathology on CAG. Furthermore, we found no evidence of rsFC moderating the relationship between pathology and CAG.

**Conclusion:**

Our results do not support the initial hypothesis, which predicted hyperconnectivity in distinct brain regions with high pathology. This could be due to relatively low overall pathology burden, consistent with an early preclinical stage of potential disease progression. This interpretation is further supported by the absence of significant associations between pathology or rsFC strength and memory performance. We identified lower rsFC strength in two distinct connections with higher blood‐based pathology. These alterations were not observed in the A‐T‐ subcohort, suggesting it to be an early marker differentiating pathological from healthy aging.